# Educational roles impact burnout in paediatric undergraduate medical educators

**DOI:** 10.1111/tct.13549

**Published:** 2022-11-06

**Authors:** Jessica L. Fealy, Angela Punnett, Heather L. Burrows, Ada M. Fenick

**Affiliations:** ^1^ University of Michigan Medical School Ann Arbor Michigan USA; ^2^ SickKids, Temerty Faculty of Medicine University of Toronto Toronto Canada; ^3^ Yale School of Medicine New Haven Connecticut USA

## Abstract

**Background:**

Physician burnout impacts all levels of medical education and has a relatively unknown impact on those responsible for medical student education, particularly in paediatrics. This study examines the prevalence of burnout among paediatric undergraduate medical educators and explores the impact of roles in medical education on medical educator burnout.

**Methods:**

This cross‐sectional mixed‐methods study utilised a binational survey of paediatricians involved in undergraduate medical education. Respondents answered demographics, standardised questions about burnout and attitudes towards students, and an open‐ended probe about interactions between medical student education and wellness.

**Findings:**

Of 445 possible, 120 (26.9%) responded to demographic and burnout questions. Of these, 23.3% endorsed burnout, 21.7% high emotional exhaustion (EE) and 10.8% high depersonalisation (DP). High levels of student‐related burnout symptoms were reported by fewer than 5% of respondents and were correlated with overall EE and DP. Content analysis revealed four emergent themes: positive effect of student‐related role, need to balance medical education and clinical roles, impact of protected time and medical education‐related autonomy on educator well‐being, and the burden of the administrative portion of educational roles.

**Discussion:**

Participating paediatric educators had low rates of burnout compared with paediatricians as a whole in prior studies. The vast majority found working with students rewarding and described the overall positive impact of their medical education role on wellness.

**Conclusion:**

Physician involvement in rewarding non‐clinical activities may improve their overall well‐being. Providing dedicated time for these activities may ameliorate the difficulty that many medical educators described in balancing their clinical and educational roles. Future studies should continue to explore how we can better support medical educators and the impact of this support on burnout.

## 1 INTRODUCTION

Burnout, the constellation of emotional exhaustion (EE) and depersonalisation (DP), challenges medical practice and pervades medical education.^1^ Burnout increases risk for physician mental health issues, substance abuse and suicide and contributes to adverse patient outcomes.^2^ Physician burnout adversely impacts specialty recruitment, physician turnover, trainee mental health and trainee well‐being.^2^, ^3^ In 2017, physicians had a 39.8% prevalence of burnout compared with 28.1% of the US working population on a two‐item burnout measure evaluating EE and DP.^1^ Of physician respondents, 36.4% reported symptoms of EE and 18% reported symptoms of DP, compared with the working population (24.8% EE and 13.5% DP).^1^ Compared with other physician specialties, paediatric generalists (40%) have similar burnout rates; only 33% of paediatric subspecialists reported at least one symptom of burnout.^1^ Although rates vary among different studies, female respondents, respondents with non‐White minority status and older physicians report higher levels of burnout, especially EE, compared with younger, White, male peers.^1^, ^4^

Practical solutions to address physician burnout remain elusive and difficult to implement.^5^, ^6^ Many hospitals have implemented wellness programmes that direct initiatives towards physicians, such as mindfulness, yoga and mental health support.^5^, ^6^ Such initiatives often fail to address the root causes of burnout, which may be better ameliorated utilising systemic initiatives such as improving teamwork, addressing moral injury/distress, increasing autonomy and modifying scheduling or workload.^5^, ^6^, ^7^ A 2017 systematic review and meta‐analysis indicated that systemic initiatives were more successful than physician‐directed initiatives; identifying protective activities on this level offers additional practical solutions to decrease physician burnout.^5^, ^6^

Practical solutions to address physician burnout remain elusive and difficult to implement.^5^

Studies of physician burnout suggest that involvement in self‐determined meaningful activities can be protective.^8^ The Mayo collaborative described overall rates of physician burnout exceeding 50%, compared with 30% in academic physicians who were provided with 10%–20% work time to pursue their passions, and recommended that health care systems provide at least 1 half‐day weekly to engage in self‐directed, meaningful work.^8^, ^9^ Internal medicine clerkship directors (CDs) report lower burnout than other internal medicine physicians as a whole. Despite 9% of surveyed internal medicine physicians in the Mayo study identifying education as the most meaningful aspect of their career, more than 50% of internal medicine CDs experienced burnout that appeared to impact their attitudes towards students.^9^ Internal medicine programme directors and psychiatry CDs both showed lower rates of burnout when compared with national studies of same‐specialty peers.^9^, ^10^ No studies to date describe burnout among paediatric educators. This study aims to examine the prevalence of burnout in paediatric CDs and the protective effect, if any, of working with students. In addition, we explore the impact of demographics, dedicated educational full‐time equivalent (FTE) and the impact of educational roles on educator well‐being.

Studies of physician burnout suggest that involvement in self‐determined meaningful activities can be protective.

## 2 METHODS

This cross‐sectional study surveyed paediatricians involved in undergraduate medical education (UME). The study population was composed of members of COMSEP (Council on Medical Student Education in Pediatrics), the US and Canadian professional organisation for academic paediatricians who supervise medical student education. COMSEP performs an anonymous annual survey of members, which is electronically sent to all COMSEP members once and response is not required or compensated. The 2019 survey included questions designed to elicit information about individuals' demographics, employment status and levels of burnout. Demographic questions included gender, age, ethnicity and geographic region. Members who self‐identified as African‐American, Latinx, Asian or ‘other’ were classified as ‘non‐White’ for demographic purposes. Although the formal ‘Under‐represented in Medicine’ designation is more stringent than this, the survey team felt it was important to explore the impact of being ‘non‐White’ because in the current landscape of microaggressions, it often matters less about how one self‐identifies and more about how others *think* you identify. Racism still impacts Asian physicians and physicians who self‐identify as ‘other’, even if they may not be specifically ‘under‐represented’ based on their proportion of the medical workforce compared with their proportion of the general population. Employment questions included specialisation, academic rank, role in UME (CD or other medical student educator, e.g., associate CD, site director and dean's office), class size and percentage of employment allocated (FTE) to UME. Comparative demographic data are unavailable for the COMSEP membership as a whole at the time of the survey and, thus, we were unable to determine if the sample was fully representative of the broader membership. The study was deemed exempt by the University of Michigan IRB; there was no independent funding for this project. Notably, this survey was performed prior to the ongoing SARS‐COVID‐19 pandemic.

Burnout questions in the survey included a validated two‐question scale originating in the Maslach inventory—an item for DP, ‘I have become more callous towards people since I took this job’, and an item for EE, ‘I feel burned out from my work’.^9^ The two‐item summative score is less cumbersome than the full Maslach Burnout Inventory (MBI) and correlates strongly with the EE and DP domains of burnout measured by the full MBI.^1^, ^11^ The two‐item MBI has been validated in several studies of physicians and medical trainees with positive predictive values of the single‐item thresholds for high levels of EE and DP, which are 88.2% and 89.6%, respectively.^11^, ^12^ The combined score had 85%–87% sensitivity and 84%–85% specificity for burnout compared with the full MBI, and there was consistency across the 2 years in one of these studies.^13^ We also included two questions, which Dyrbye et al. found to be associated with a higher prevalence of burnout in internal medicine CDs regarding student relationships modelled after questions on the MBI; the first, ‘I don't really care what happens to some of my students’, associated with DP towards students and the second, ‘Working with students directly puts too much stress on me’, associated with EE concerning students.^3^ The research team added two additional questions to further assess the emotional tenor towards students, ‘I feel guilty about my attitude at work towards students’ and ‘I find working with students rewarding’. These questions were generated by the COMSEP Wellness Collaborative (working group), piloted on its members and used the same MBI response options. The question regarding guilt was added because of the high rates of guilt associated with burnout as demonstrated in Baer et al.'s study examining burnout in paediatric residents, showing that residents with symptoms of burnout were three to nine times more likely to self‐report suboptimal patient care attitudes and behaviours, including, ‘felt guilty from how I treated a patient from a humanitarian standpoint’.^14^ In highly empathetic individuals, feelings of guilt increase the risk for internalised/moral distress and thus may increase risk for presence or development of burnout.^15^ We included a question about finding working with students rewarding to explore our hypothesis that this reward may mitigate burnout. All burnout questions (Box 1) were scored 0–6 using a 7‐point Likert scale of frequency from *never* to *every day*; the question regarding reward working with students was reverse scored.

Box 1 **Burnout questions**

1. Emotional exhaustion (EE): ‘I feel burned out from my work.’
2. Depersonalisation (DP): ‘I have become more callous towards people since I took this job.’
3. Emotional exhaustion towards students (EES): ‘Working with students directly puts too much stress on me.’
4. Depersonalisation towards students (DPS): ‘I don't really care what happens to some of my students.’
5. Feels guilty about students (FGS): ‘I feel guilty about my attitude at work towards students.’
6. Finds working with students rewarding (WSR): ‘I find working with students rewarding.’

When evaluating burnout literature, some studies report symptoms of burnout as either a logical conjunction (Venn ‘and’) of EE and DP or a logical disjunction (Venn ‘or’) of EE or DP.^16^ For our study, all questions were analysed independently with a positive score defined as reporting symptoms at least weekly (score ≥ 4), other than the question regarding feelings of reward from working with students, which was reverse scored. Burnout was defined as either EE or DP positive for symptoms at least weekly, using the disjunction, as in the study on paediatric residents by Kemper et al.^13^

Respondents were also asked to expand on the role of medical students in their wellness through typed response to an open‐ended prompt, ‘How does your role in medical education affect your wellness? What is the impact of wellness, or lack thereof, on your professional role in medical education?’

How does your role in medical education affect your wellness?

Analysis of quantitative data was performed using SAS 9.4. We used the Spearman correlations to examine the correlations among the different burnout measures. When the demographic variable was continuous, Student's t‐test and the Mann–Whitney U‐test were used when appropriate for statistical comparison. When the demographic variable was categorical and the subgroups were large enough, we used a chi‐squared test for analysis, and when there were smaller cells (in the cases of ethnicity, region, academic rank and specialty), we used the Fisher exact test. We used the Spearman correlations to examine the correlation between dedicated educational FTE and the feelings of burnout.

A thematic and sentiment analysis of responses to the single open‐ended question was performed by two authors (both medical educators of students and COMSEP members) independently reading through the responses and assigning both codes and valences (positive, neutral/mixed or negative in student effect on burnout) to each statement. Responses were iteratively reviewed, and differences were reconciled through review and discussion. Themes were identified and member checking was conducted by sharing themes and example statements with a subset of the COMSEP membership attending a 2021 annual conference session on wellness. The 15 members who participated approved the themes as presented in the 30‐minute review and discussion. Given the anonymous nature of the survey, we cannot ascertain if the members participating in the member checking had also completed the survey.

## 3 RESULTS

Of 445 COMSEP members who received the 2019 annual survey, 120 completed both the demographic and the burnout sections (26.9% response rate). The majority of respondents (61%) identified as White/Non‐Hispanic; 67% identified as female (Table 1). Most respondents were either assistant or associate professors, approximately one third were CDs, two‐thirds were other medical student educators, about half were generalists and half were subspecialists, and, as a group, they averaged 0.3 FTE for their educational role. The Spearman correlation test was used to examine any relationship between demographic variables, and the only finding of significance was that age and academic rank were positively, though weakly, correlated (rho = 0.642).

**TABLE 1:** Demographics of respondents, n = 120

Demographic	n (%)
Gender	
Female	80 (66.7)
Male	37 (30.8)
No response	3 (2.5)
Age	
≤40 years	42 (35.0)
41–50 years	40 (33.3)
≥51 years	34 (28.3)
No response	4 (3.3)
Ethnicity	
Non‐White	14 (11.7)
White, Non‐Hispanic	73 (60.8)
No response	33 (27.5)
Region	
Northeast	26 (21.7)
Midwest	29 (24.2)
South	45 (37.5)
West	15 (12.5)
Canada	5 (4.2)
Academic rank	
Instructor	3 (2.5)
Assistant professor	49 (40.8)
Associate professor	40 (33.3)
Professor	24 (20.0)
No response	4 (3.3)
Specialty	
Generalist	54 (45.0)
Subspecialty	52 (43.3)
No response	14 (11.7)
Role	
Clerkship director	42 (35.0)
Other medical student educator	78 (65.0)
Class size^a^	140 (61.1)
Full‐time equivalent^a^	0.3 (0.16)

^a^ Mean (SD).

**FIGURE 1:**
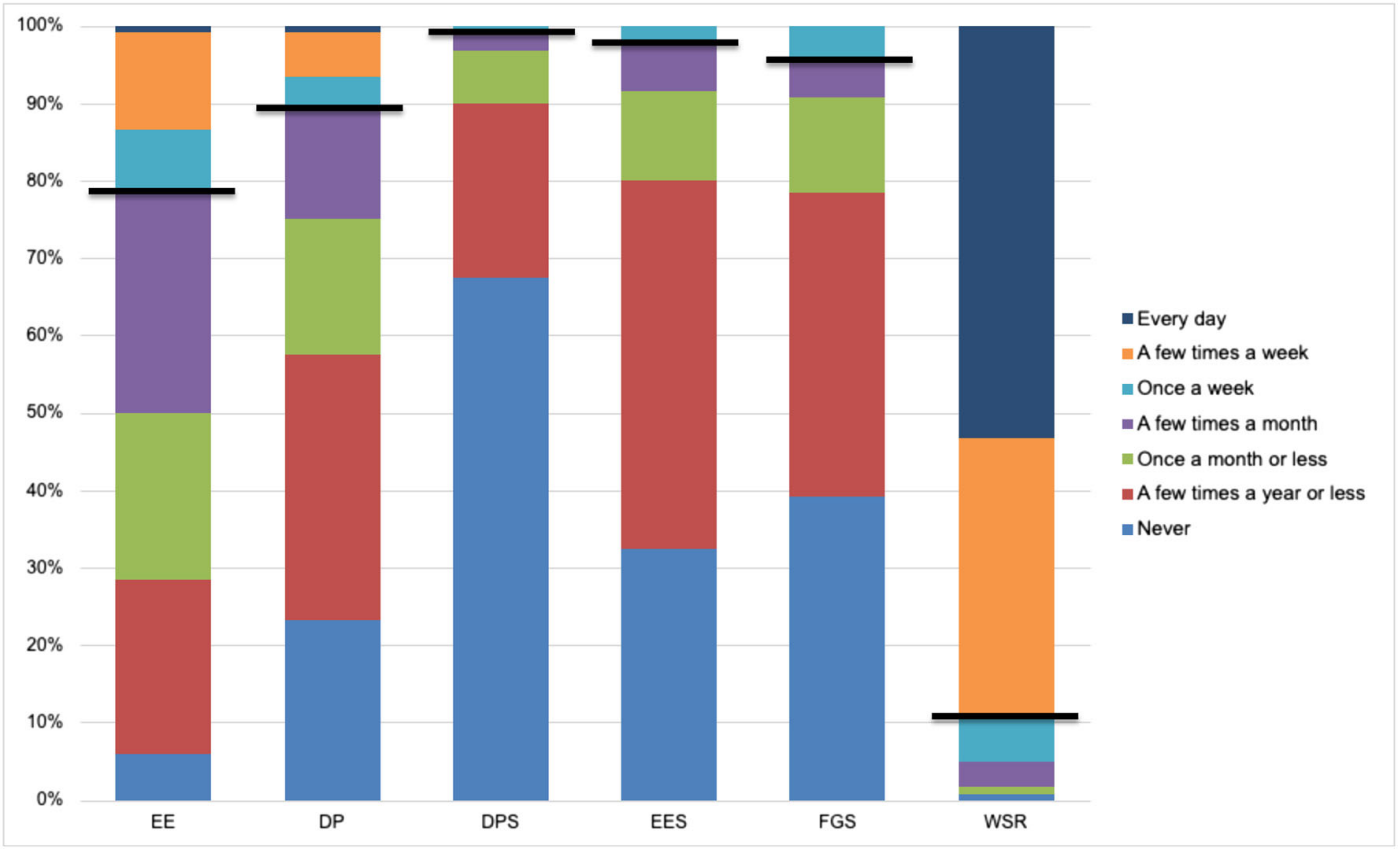
Response to burnout questions by percentage of respondents. DP, depersonalisation; DPS, depersonalisation towards students; EE, emotional exhaustion; EES, emotional exhaustion towards students; FGS, feels guilty about students; WSR, finds working with students rewarding

Burnout was endorsed by 23.3% of the respondents. High EE was reported by 21.7% of the cohort, and DP was reported by 10.8% of the cohort (Figure 1). Student‐related burnout symptoms were less prevalent, as only 2.5% had a positive EE regarding students and 0.8% had a positive DP regarding students. Feelings of guilt regarding their attitude towards students were reported by 4.2% of respondents, whereas, notably, 95% of respondents found working with students rewarding.

Strong correlations emerged among the burnout questions (Table 2). EE was associated with DP (rho = 0.643, p ≤ 0.0001). Overall EE and DP were both associated with EE towards students, DP towards students and feelings of guilt regarding attitude towards students, but there was no correlation with finding working with students rewarding because paediatric educators reported working with students rewarding regardless of other burnout symptoms. Examining student measures, EE towards students, DP towards students and feelings of guilt regarding attitude towards students were all correlated with each other, and feelings of reward towards working with students was inversely correlated with the other three student‐related measures.

**TABLE 2:** Correlations among burnout responses

	Emotional exhaustion	Depersonalisation	Emotional exhaustion towards students	Depersonalisation towards students	Feels guilty about students
Emotional exhaustion					
Depersonalisation	0.643 <0.0001				
Emotional exhaustion towards students	0.256 <0.01	0.297 <0.001			
Depersonalisation towards students	0.187 <0.05	0.377 <0.0001	0.392 <0.0001		
Feels guilty about students	0.281 <0.01	0.436 <0.0001	0.475 <0.0001	0.473 <0.0001	
Finds working with students rewarding	−0.130 NS	−0.169 NS	−0.184 <0.05	−0.321 <0.001	−0.25996 <0.005

*Note*: Data are presented as the Spearman correlation, given as rho, p. Grey indicates repeat or self‐correlation.

Abbreviation: NS, not significant.

There were no statistically significant associations between demographics of gender, age, ethnicity, region, academic rank, subspecialisation, medical education role or FTE and the individual measures of EE and DP (data not shown).

In content analysis, narrative response valences were coded as 50% positive, 28% neutral/mixed and 22% negative, with respect to medical education roles and their impact on wellness. Themes that emerged included (Table 3)

1. positive effect of student‐related role—most respondents found working with students rewarding, regardless of their own feelings of burnout;
2. need to balance medical education and clinical roles—many respondents noted the pressure to balance multiple roles and the time and effort involved;
3. impact of protected time and medical education‐related autonomy on educator well‐being—many respondents noted it was easier to balance these roles if they had dedicated educational time or administrative support; and
4. the burden of the administrative portion of educational roles—respondents with administrative support found it easier to balance their educational and clinical responsibilities.

**TABLE 3:** Thematic and sentiment analysis of open‐ended question

Theme	Sentiment	Illustrative statements
Positive effect of student‐related role	Positive	Students are my treatment for burnout. It is a pleasure to have different things to do and think about, and not the same thing all the time. MedEd serves this role. It is refreshing and rejuvenating. It allows for thinking about the future not only of our small patients in front of us, but of all small patients touched by anyone we teach. So, it gives hope. I find being involved in education as a positive factor in my wellness—as working with students allows for a sense of meaningful work, even if I am facing challenges/barriers/burnout in my clinical work. I find that students bring an energy and engagement to the field of medicine that is refreshing and infectious. Improves my wellness because I get so much joy and satisfaction in working with students. I think it's a positive cycle because my joy makes me a better educator.
Need to balance medical education and clinical roles	Positive	It (*role in medical education*) improves my wellness. Work in medical education offers balance with my clinical responsibilities. Working with students often helps put my clinical/administrative work into a larger perspective and keeps me grounded. If I were clinical only, I think I would feel much more burnout. Involvement in education gives me more chances to be successful and more autonomy to try new things. This is not valued at all in my clinical setting.
	Negative	Takes me away from patients, which often impacts my ability to care for them, which impacts my well‐being.
Protected time and autonomy related to medical education impact well‐being	Positive	Wellness depends on the support I get from the deans of the school, which has been very helpful. Improves wellness—gives me a role and purpose in institution as well as breaks up week and activities and gives more flexibility for work–life balance.
	Negative	Lots of work for students with almost no protected time adds to stress and decreases wellness. It adds more work to my plate along with my clinical responsibilities, which can at times be challenging and/or difficult to balance. There are lots of responsibilities that do not have adequate time to complete at the level I know I could if I had appropriate dedicated time. This means that I am using my personal time to complete, which can increase my perceived stress level. I feel guilty when I am not working on school projects when I am at home.
The administrative burden of the student role decreases satisfaction	Neutral/mixed	Direct teaching is a blast; the administrative burden is problematic. Mentoring activities restore while administrative activities deplete. Making an impact in a learner's life rejuvenates me. It's the paperwork, the grading, the grade appeals etc. that get me down. While medical education can be exciting and rejuvenating, at times, with minimal support from admins, it can be overwhelming. Interactions with students affect it (*wellness*) positively. The energy that comes with my love of teaching and feeling their interest and engagement helps my wellness. The time all of the administrative details take and the seemingly endless tasks involved that expand into my personal time and hand *(sic)* over my head have a large negative impact on my wellness. The good still very much outweighs the negative.

Themes that emerged included

1. positive effect of student‐related role;
2. need to balance medical education and clinical roles;
3. impact of protected time and medical education‐related autonomy; and
4. the burden of the administrative portion of educational roles.

Member checking resulted in universal thematic approval and suggested no supplementary elements.

## 4 DISCUSSION

Paediatric medical educators in this study had a low rate of burnout (23.3%) in comparison with published studies for paediatricians as a whole (33% for subspecialists and 40% for general paediatricians).^1^ Respondents described the overall positive impact of their medical education role on their wellness and 95% found working with students rewarding. By contrast with other studies, we did not find significant associations between individual symptoms of burnout and gender, self‐identified race/ethnicity or age.

Paediatric medical educators in this study had a low rate of burnout (23.3%) in comparison with published studies for paediatricians as a whole (33% for subspecialists and 40% for general paediatricians). Respondents described the overall positive impact of their medical education role on their wellness and 95% found working with students rewarding.

Decreased burnout has been documented among educators previously. Dyrbye et al. found that 46% of internal medicine CDs reported high EE and 41% had high DP, less than the 50% of internists who reported burnout.^1^, ^3^ Psychiatry CDs similarly had less burnout than psychiatrists in general, with 14%–22% of psychiatry CDs reporting some level of burnout, compared with approximately 40% in their same‐specialty peers.^1^, ^10^ The general finding that medical educators report lower rates of burnout in standardised surveys may be explained by the educators in our survey, who, when asked about the relationship between students and burnout, noted the positive effect of the student‐related role and overwhelmingly found working with students rewarding. However, many respondents also identified challenges in finding balance between their clinical and educational roles. Many respondents appreciated the increased autonomy related to the educational role, counterbalanced chiefly by the administrative burden, noting that dedicated time and administrative support helped make achieving balance easier.

Educators noted the positive effect of the student‐related role and overwhelmingly found working with students rewarding.

This echoes a Delphi‐method study in which a multispecialty group of clerkship educators deemed that critical resources, including dedicated time and administrative assistance, were necessary to perform the essential elements of medical education.^17^ Although the internal medicine CDs had triple the odds of burnout if they had less than 10 hours per week of dedicated time for the clerkship, FTE was not significantly related to burnout in our survey, though comments did indicate that lack of time to handle the clerkship was a source of stress, which may be a risk factor for later burnout.^3^ The themes that were identified by participants also echo many of the external and individual factors affecting clinician well‐being and resilience described in the National Academy of Medicine (NAM) conceptual model, particularly in categories of Health Care Role, Personal Factors, Organisational Factors and Learning/Practice Environment.^18^

Strong correlations emerged between the burnout questions and three of four questions regarding attitudes towards students: EE towards students, DP towards students and feelings of guilt related to working with students, which were all highly correlated with each other. In the prior study by Dyrbye et al., the questions about DP towards students and EE towards students were indicative of higher odds of EE or DP^3^; the novel question of feelings of guilt related to students was not previously examined. Taken together, the two studies may indicate that these three questions may be useful in future assessments of burnout in educators. In contrast, although 95% of educators found working with students rewarding is positive, the discriminative ability of this question was limited.

Our study is subject to several limitations, including a low response rate of 27% of possible respondents, affecting the quantitative portion of the study. People experiencing burnout may be less likely to complete surveys, and it is also possible that generalised burnout, or that related to students, may contribute to attrition in medical education roles, affecting the population of educators involved in education/COMSEP membership; both factors may contribute to lower rates of burnout in the study group. Because of the lack of accurate data on the overall membership of COMSEP, we are unable to determine if the present sample is representative of COMSEP membership or of all US/Canadian paediatric medical educators. Because burnout rates are known to be higher in physicians who are non‐White, the low diversity of respondents is also a limitation; if this lack of diversity is accurate, it demonstrates that we have work to do in improving the diversity of leadership in medical education as a means to widening the pathway and providing mentorship to students under‐represented in medicine.

In 2017, 61% of the general adult working population reported satisfaction with work–life integration; however, only 40% of physicians did so and general paediatricians had approximately 50% satisfaction with work–life integration.^1^ Future population‐wide studies of physician employment may attempt to correlate job characteristics of subpopulations of physicians, such as educational employment and interpersonal orientation, with burnout and job satisfaction. The authors are mindful of the impact sampling bias may have on our results and are contemplating more expansive ways of monitoring educator burnout/well‐being. More aspirationally, future studies may allow us to learn how we capitalise on and promote the meaningful work of education to better support medical educators. The impact on burnout of gender, non‐White identity and age should be reviewed as the diversity of medical educators increases. Lastly, it may also be useful to examine the impact of the COVID‐19 pandemic on the well‐being of paediatric medical educators and paediatricians in general. The NAM model emphasises the impact of clinician well‐being on patient well‐being and could be extrapolated to examine the impact of educator well‐being on learner well‐being.^18^

## 5 CONCLUSION

Paediatric medical educators report fewer burnout symptoms than their same‐specialty peers and other specialty educators. Working with students was rewarding and student‐related roles had positive impact, regardless of personal burnout symptomatology. Asking educators about EE towards students, feelings of DP towards students and feelings of guilt around working with students may help evaluate burnout. The positive, and perhaps protective, impact that working with students has on the wellness of the physician educators may support provision of protected time for physicians to dedicate to rewarding non‐clinical activities to improve their sense of autonomy and overall sense of well‐being. Respondents identified the difficulty in balancing their medical education and clinical roles and that achieving this was easier with dedicated educational time and administrative support. Similar to graduate medical education (GME) faculty, UME faculty would benefit from dedicated FTE and administrative support for their clerkship roles.^19^ Alleviating some of this role balancing stress may mitigate burnout and may help programmes and department leaders to recruit, support and retain educators in paediatrics.

UME faculty would benefit from dedicated FTE and administrative support for their clerkship roles.

## CONFLICT OF INTEREST

The authors have no conflict of interest to disclose.

## ETHICAL APPROVAL

The study was deemed exempt by the University of Michigan IRB. All participants consent to the sharing and submission of their responses for publication when they complete the COMSEP annual survey.
